# Comorbid Autoimmune Diseases in Patients With Myasthenia Gravis: A Retrospective Cross-Sectional Study of a Chinese Cohort

**DOI:** 10.3389/fneur.2021.790941

**Published:** 2021-11-22

**Authors:** Jianquan Shi, Xiao Huan, Lei Zhou, Jianying Xi, Jie Song, Ying Wang, Sushan Luo, Chongbo Zhao

**Affiliations:** ^1^Department of Neurology, Nanjing First Hospital, Nanjing Medical University, Nanjing, China; ^2^Department of Neurology, Huashan Hospital Fudan University, Shanghai, China; ^3^National Center for Neurological Disorders, Shanghai, China; ^4^Department of Pharmacy, Huashan Hospital, Fudan University, Shanghai, China

**Keywords:** myasthenia gravis, autoimmune diseases, comorbidities, percentage, clinical characteristics

## Abstract

**Introduction:** The phenomenon of coexisting autoimmune diseases (ADs) in patients with myasthenia gravis (MG) has attracted considerable attention. However, few studies have investigated the burden and potential clinical associations of ADs in Chinese MG cohorts.

**Methods:** In this retrospective cross-sectional study, we reviewed the records of 1,132 patients with MG who were admitted to Huashan Hospital Fudan University from August 2013 to August 2020. Patients were excluded if they had incomplete medical records (*n* = 336).

**Results:** Comorbid ADs were found in 92 of 796 Chinese patients with MG (11.6%), among which, hyperthyroidism (6.7%), hypothyrosis (2.6%), and vitiligo (0.8%) were predominant. Patients with MG with ADs were predominantly female, younger at the onset of MG symptoms, and had a lower frequency of thymoma. Compared to the general population, we found a significantly higher percentage of hyperthyroidism (8.5-fold increase, *p* < 0.001), hypothyrosis (2.6-fold increase, *p* < 0.001), vitiligo (1.3-fold increase, *p* < 0.001), rheumatoid arthritis (1.4-fold increase, *p* < 0.001), immune thrombocytopenic purpura (193.1-fold increase, *p* < 0.001), autoimmune hemolytic anemia (7.4-fold increase, *p* < 0.001), autoimmune hepatitis (5.1-fold increase, *p* < 0.001), and polymyositis (11.5-fold increase, *p* < 0.001) in patients with MG with ADs. Patients with MG with ADs presented a lower proportion of previous history of MC (0 vs. 5.6%, *p* < 0.05) than those without ADs. The proportion of MGFA Class I at onset in patients with MG with ADs was significantly higher than that in patients with MG without ADs (77.0 vs. 52.7%, *p* < 0.05). The proportion of MuSK-positive in patients with MG with ADs was significantly lower than that in patients with MG without ADs (0 vs. 4.8%, *p* < 0.05).

**Conclusion:** In conclusion, we observed a higher frequency of concurrent ADs in a Chinese MG cohort. Furthermore, MG combined with ADs tended to have mild clinical presentation.

## Introduction

Myasthenia gravis (MG) is a rare autoimmune disorder of the neuromuscular junction, which is characterized by fatigable weakness in extraocular muscles, limbs, and even bulbar muscles (1). The disorder is typically mediated by antibodies against acetylcholine receptor (AChR) or other proteins located at the neuromuscular junction, including muscle-specific tyrosine kinase (MuSK) and lipoprotein receptor-related protein 4 (2). The incidence of MG ranges from 0.3 to 2.8 per 100,000 worldwide, which varies with age, sex, and ethnic groups (3, 4).

Similar to other autoimmune diseases (ADs), genetic factors contribute to the susceptibility of developing MG (5). Mounting evidence has demonstrated common genetic signals in many ADs, suggesting the possibility of shared common pathogenetic mechanisms (6). Several studies from western countries and Japan have suggested that ~13% of patients with MG also have other ADs (7–10). The most common AD in patients with MG is autoimmune thyroid disease, followed by systemic lupus erythematosus and rheumatoid arthritis (11). MG in association with ADs often has mild clinical expression (11). However, it remains unclear whether there are clinically significant differences in Chinese patients with MG with and without ADs. Therefore, we investigated the percentages of comorbid ADs in a Chinese MG cohort. Furthermore, we compared the clinical characteristics between patients with MG with and without ADs.

## Methods

### Study Design and Patient Recruitment

Through our single-center registration database, we reviewed the records of all patients with MG who were admitted to Huashan Hospital Fudan University from August 2013 to August 2020. The diagnosis of MG was based on clinical symptoms and at least one of the following specific tests: objective clinical response to neostigmine test, seropositivity for anti-AChR/anti-MuSK antibody, or significant decremental response on 3 Hz repetitive nerve stimulation (12, 13). Other diseases that mimic MG were excluded, including Lambert-Eaton syndrome, motor neuron disease, and congenital myasthenic syndrome.

ADs were identified if they were included in the list defined by Hayter and Cook, with the addition of psoriasis (14–16). Diagnoses of ADs were based on the clinical manifestations, laboratory test (including specific antibodies), biopsy results (if required), and reference to the diagnostic criteria of ADs by the corresponding specialists.

This retrospective cohort study was approved by the Institutional Review Board of Huashan Hospital Fudan University. Written informed consent was obtained from each study participant.

### Data Collection

Clinical data were collected from the recruited patients, including age at onset, duration, sex, thymoma concurrence, thymectomy, history of myasthenic crisis (MC), family history of ADs, history of allergic diseases, family history of allergic diseases, history of malignancies, Myasthenia Gravis Foundation of America (MGFA) Class at onset, serum antibody status, and the presence of comorbid ADs.

### Statistical Analysis

Statistical analyses were performed using IBM SPSS version 21.0 (SPSS Inc., Chicago, IL, USA). The normality of continuous variables was tested by Shapiro–Wilks test. Continuous variables that followed a normal distribution are presented as the mean ± standard deviation. Categorical variables are expressed as frequencies (percentages). The chi-square test for specified proportions was used to compare the percentages of various ADs in patients with MG to the corresponding prevalence in the general population. The most recent prevalence data for each AD were selected for comparison. Data obtained from the Chinese population were used when available. The prevalence of other countries was used when Chinese prevalence data were unavailable. Independent sample *t*-test and chi-square test were used to compare the differences between the two groups as appropriate (MG with ADs vs. MG without ADs; MG with 1 AD vs. MG with ≥2 ADs). A two-tailed *p* < 0.05 was considered significant.

## Results

### Clinical Characteristics of MG Cohort

**Figure 1:**
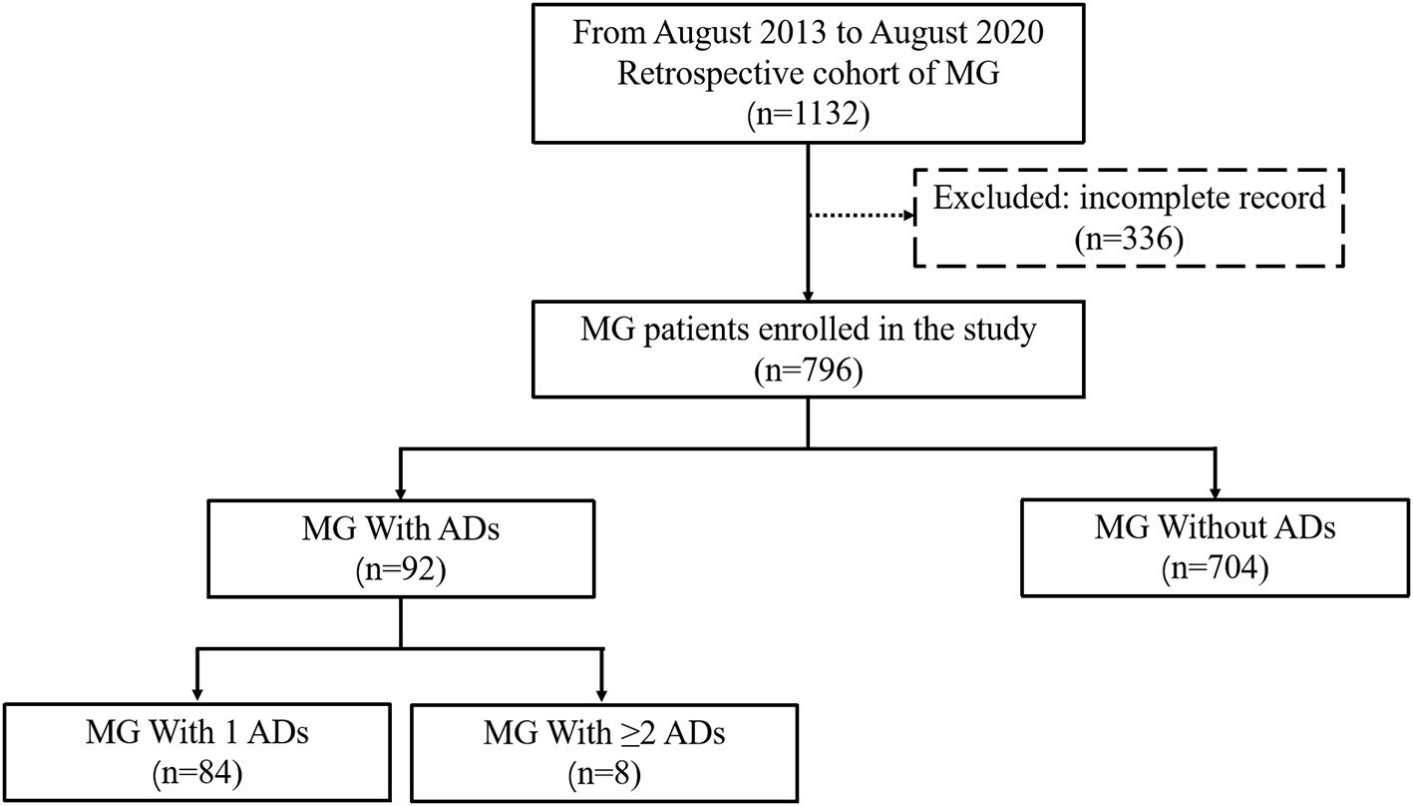
Flow diagram of the study design. A total of 796 patients with MG were enrolled in the study, including 704 patients with MG without ADs and 92 with ADs (84 with 1 ADs and 8 with ≥2 ADs). MG, Myasthenia gravis; Ads, Autoimmune diseases.

The database comprised 1,132 steroid or immunosuppressant naïve patients who were diagnosed with MG during the study period. Patients were excluded if they had incomplete medical records (*n* = 336). Ultimately, 796 patients with MG were enrolled in the study, including 704 patients without ADs and 92 patients with ADs (84 with 1 ADs and 8 with ≥2 ADs) (Figure 1).

The clinical characteristics of patients with MG are presented (Table 1). Among the 796 patients, 454 (57.0%) were female, the average age at onset was 40.4 ± 18.1 years, and the average duration was 2.9 ± 5.6 years. Thymoma was present in 214 cases (26.9%), and thymectomy was performed in 120 cases (15.1%). As the initial symptom, ocular weakness alone (MGFA Class I) occurred in 56.4% (449/796) of patients, and 43.5% (346/796) of patients were classified into generalized type (MGFA Class II, III, IV). Only 4.3% (34/796) of patients suffered from MC. Two hundred and sixty-one cases (32.8%) had no information on antibody test. Approximately 84.9% (454/535) of patients were AChR-positive, 4.1% (22/535) of patients were MuSK-positive, and 11.0% (59/535) of patients were double-seronegative.

**Table 1:** Clinical characteristics of patients with MG.

	**No. of cases (%)**
	**(*n* = 796)**
Age at onset (years)	40.4 ± 18.1
Duration (years)	2.9 ± 5.6
Sex	
Male	342 (43.0%)
Female	454 (57.0%)
Age at onset (years)	
<50	537 (67.5%)
≥50	259 (32.5%)
Thymoma concurrence	
No	582 (73.1%)
Yes	214 (26.9%)
Thymectomy	120 (15.1%)
History of MC	
No	762 (95.7%)
Yes	34 (4.3%)
MGFA at onset	
I	449 (56.4%)
II	318 (39.9%)
III	25 (3.1%)
IV	3 (0.4%)
Unknown	1 (0.1%)
Antibody status	
AChR-positive	454 (57.0%)
MuSK-positive	22 (2.8%)
Seronegative	59 (7.4%)
Unknown	261 (32.8%)
Antibody status (*n* = 535)	
AChR-positive	454 (84.9%)
MuSK-positive	22 (4.1%)
Seronegative	59 (11.0%)

*MG, Myasthenia gravis; MC, Myasthenic crisis; MGFA, Myasthenia Gravis Foundation of America; AChR, Acetylcholine receptors; MuSK, Muscle-specific tyrosine kinase*.

### Types and Percentages of Comorbid ADs in MG Cohort

**Figure 2:**
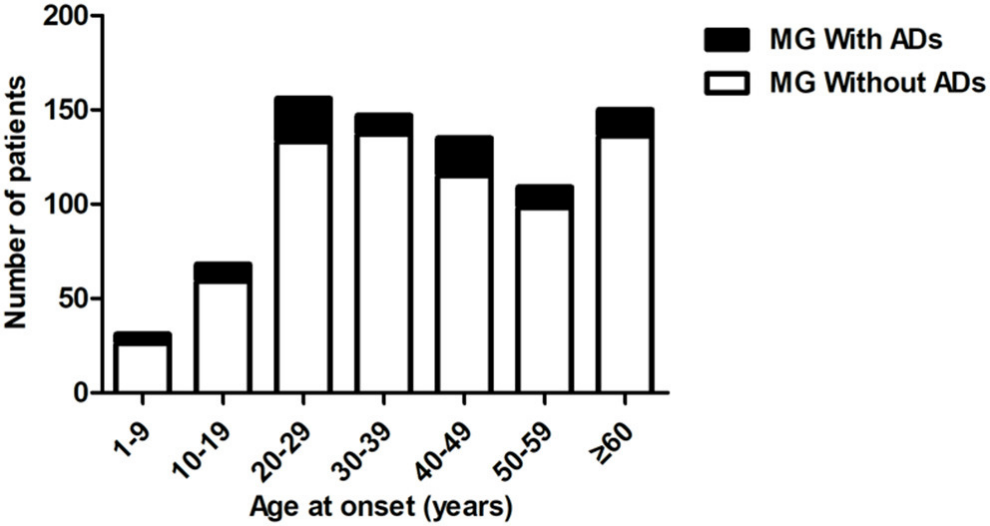
Percentage of comorbid ADs by age at onset. ADs, Autoimmune diseases.

Ninety-two patients (59 females and 33 females, 11.6%) with MG had ≥1 comorbid ADs. The frequencies of overall ADs in our cohort were higher than the background prevalence of the population (total patients: 11.6 vs. 5.0%, male patients: 9.6 vs. 3.0%, female patients: 13.0 vs. 7.1%) (15). The percentages of comorbid ADs varied among different onset age groups (from 6.8 to 16.1%, Figure 2). The percentages of comorbid ADs in patients with early onset MG (67/537, 12.5%) were higher than those in patients with late-onset MG (25/259, 9.7%). The percentage of comorbid ADs in patients with MG without thymoma (75/582, 12.9%) was higher than that in patients with MG with thymoma (17/214, 7.9%).

**Figure 3:**
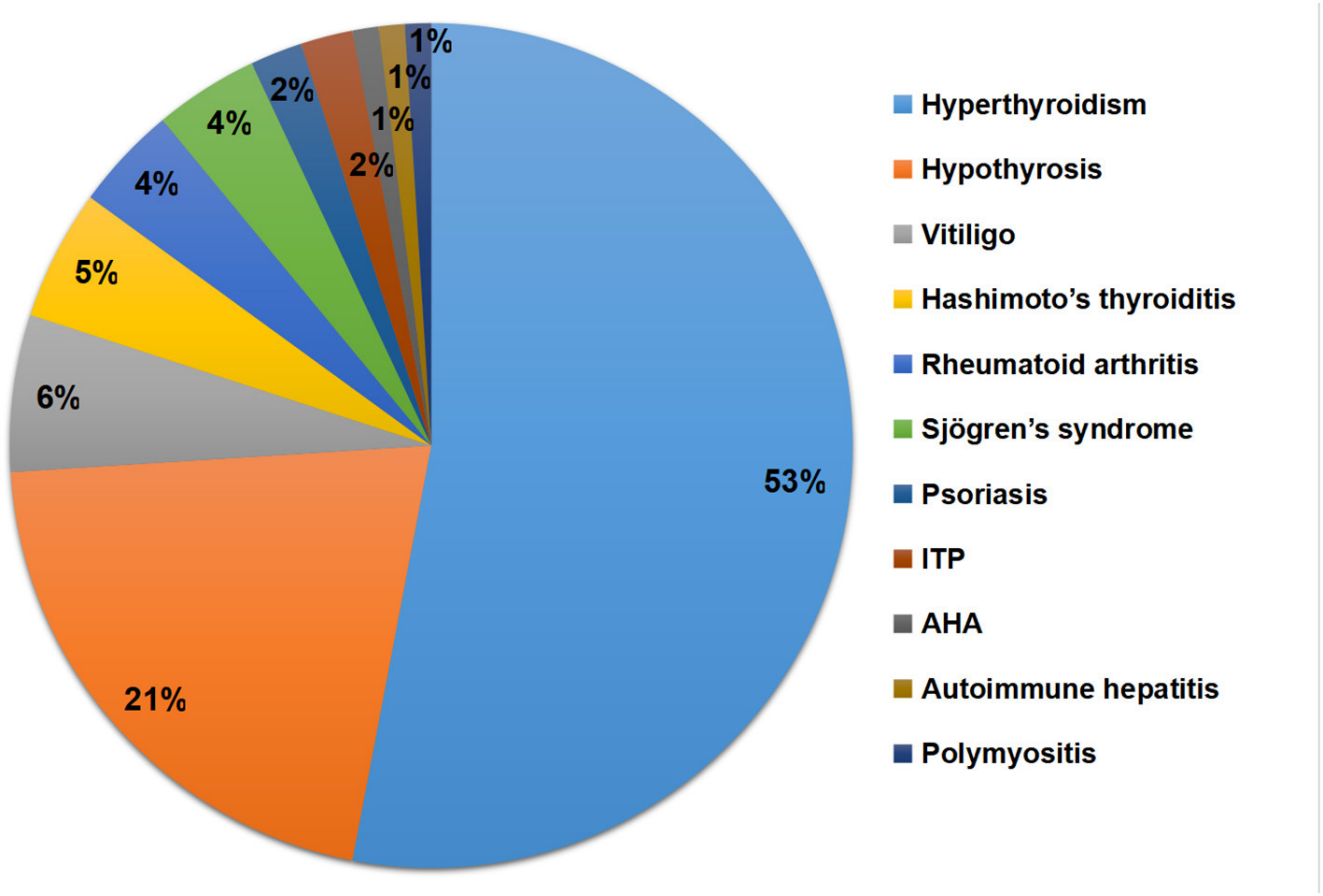
Types and percentages of concomitant ADs. ADs, Autoimmune diseases; ITP, Immune thrombocytopenic purpura; AHA, Autoimmune hemolytic anemia.

The types, frequencies, and percentages of concomitant ADs are presented in Table 2 and Figure 3. Thyroid disease was the most common comorbid AD, observed in 79 (9.9%) patients with MG. The percentage of hyperthyroidism, hypothyrosis, and Hashimoto's thyroiditis was 6.7% (*n* = 53), 2.6% (*n* = 21), and 0.6% (*n* = 5), respectively. Other types of ADs included vitiligo (*n* = 6, 0.8%), rheumatoid arthritis (*n* = 4, 0.5%), Sjögren's syndrome (*n* = 4, 0.5%), psoriasis (*n* = 2, 0.3%), idiopathic thrombocytopenic purpura (*n* = 2, 0.3%), autoimmune hemolytic anemia (*n* = 1, 0.1%), autoimmune hepatitis (*n* = 1, 0.1%), and polymyositis (*n* = 1, 0.1%).

**Table 2:** Comorbid ADs in patients with MG.

	**Frequency (%) (*n* = 796)**	**Cases (extrapolated to 100,000 patients with MG)**	**Prevalence in the general population (region)**
Hyperthyroidism	53 (6.7%)	6658	0.78% (China) (17)
Hypothyrosis	21 (2.6%)	2638	1.02% (China) (17)
Vitiligo	6 (0.8%)	754	0.56% (China) (18)
Hashimoto's thyroiditis	5 (0.6%)	628	1.7% (China) (19)
Rheumatoid arthritis	4 (0.5%)	503	0.37% (China) (20)
Sjögren's syndrome	4 (0.5%)	503	0.45% (China) (20)
Psoriasis	2 (0.3%)	251	0.47% (China) (21)
ITP	2 (0.3%)	251	0.0013% (France) (22)
AHA	1 (0.1%)	126	0.017% (Denmark) (23)
Autoimmune hepatitis	1 (0.1%)	126	0.0245% (New Zealand) (24)
Polymyositis	1 (0.1%)	126	0.011% (Africa) (25)

*MG, Myasthenia gravis; ADs, Autoimmune diseases; ITP, Immune thrombocytopenic purpura; AHA, Autoimmune hemolytic anemia*.

**Figure 4:**
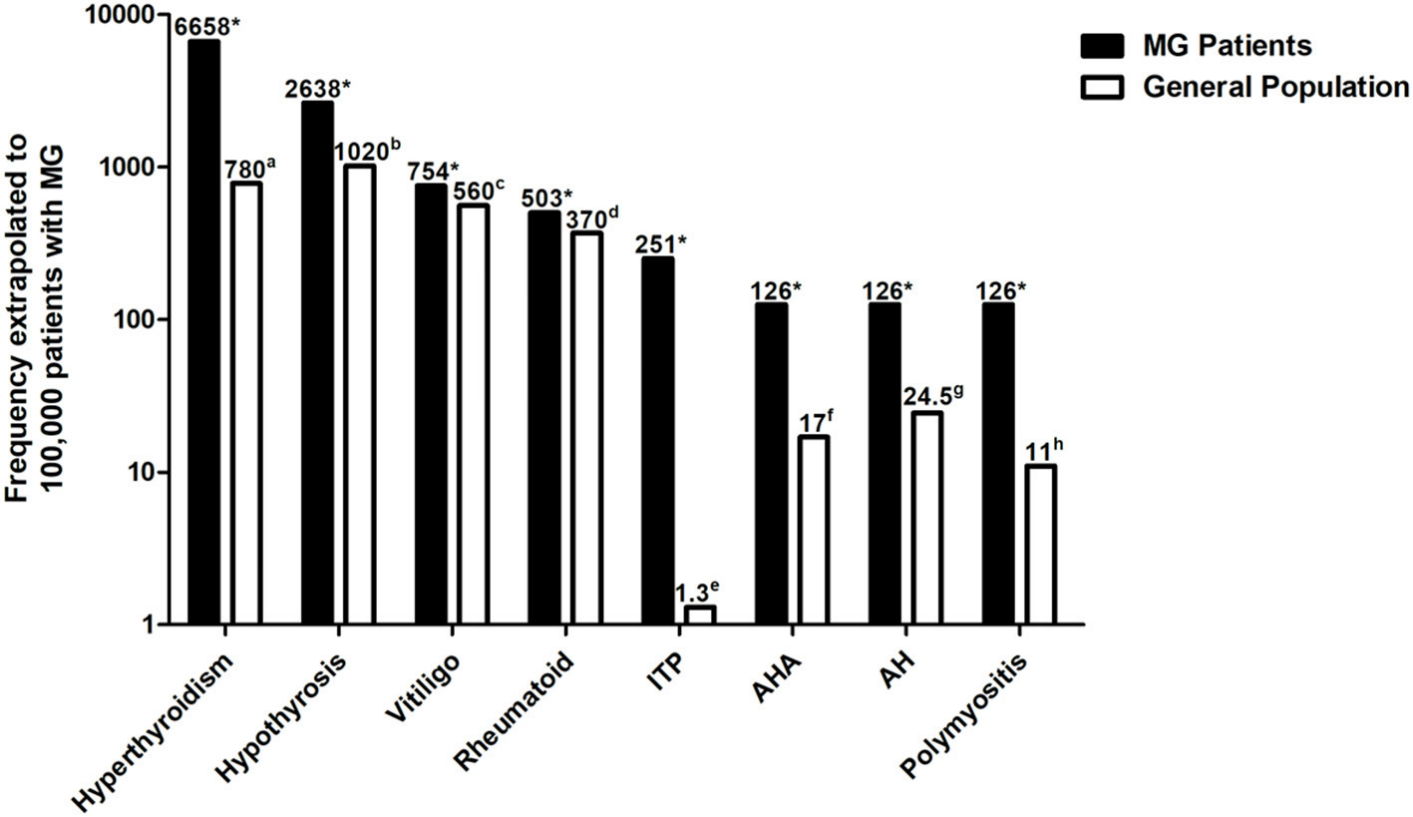
Percentage of each observed AD was extrapolated to 100,000 patients with MG and compared to the prevalence of the disease in the general population. Autoimmune diseases with a significantly higher percentage in patients with MG compared to the general population are shown. The Y-axis is in logarithmic scale. ^a^Data derived from Li et al. (17); ^b^data derived from Li et al. (17); ^c^data derived from Wang et al. (18); ^d^data derived from Xiang et al. (20); ^e^data derived from Mariotte et al. (22); ^f^data derived from Hansen et al. (23); ^g^data derived from Ngu et al. (24); ^h^data derived from Essouma et al. (25); *p < 0.05 vs. the prevalence of the disease in the general population. ADs, Autoimmune diseases; MG, Myasthenia gravis.

The percentage of each observed AD in our cohort and the prevalence of the disease in the general population are presented in Table 2. The percentage of each observed AD was extrapolated to 100,000 patients with MG and compared to the prevalence of the disease in the general population (Table 2 and Figure 4). We found a significantly higher percentage of hyperthyroidism (8.5-fold increase, *p* < 0.001) (17), hypothyrosis (2.6-fold increase, *p* < 0.001) (17), vitiligo (1.3-fold increase, *p* < 0.001) (18), rheumatoid arthritis (1.4-fold increase, *p* < 0.001) (20), immune thrombocytopenic purpura (193.1-fold increase, *p* < 0.001) (22), autoimmune hemolytic anemia (7.4-fold increase, *p* < 0.001) (23), autoimmune hepatitis (5.1-fold increase, *p* < 0.001) (24), and polymyositis (11.5-fold increase, *p* < 0.001) (25). We observed at a higher percentage of Sjögren's syndrome in patients with MG (20), although the difference did not reach statistical significance (1.1-fold increase, *p* = 0.091). Hashimoto's thyroiditis and psoriasis were observed at a lower prevalence in patients with MG (19, 21).

### Clinical Characteristics Between MG With and Without ADs

We compared the clinical characteristics between patients with MG with and without ADs. Patients with MG with ADs presented a lower proportion of previous history of MC (0 vs. 4.8%, *p* < 0.05) and a higher proportion of family history of ADs (20.7 vs. 4.5%, *p* < 0.05) than those without ADs. The proportion of MGFA Class I at onset in patients with MG with ADs was significantly higher than that in those without ADs (72.8 vs. 54.3%, *p* < 0.05). No significant differences in other clinical features were found between patients with MG with and without ADs (*p* > 0.05) (Supplementary Table 1).

We next compared the clinical characteristics between patients with MG with and without ADs for whom antibody information was available. Patients with MG with ADs presented a lower proportion of previous history of MC (0 vs. 5.6%, *p* < 0.05) and a higher proportion of family history of ADs (21.6 vs. 5.4%, *p* < 0.05) than those without ADs. The proportion of MGFA Class I at onset in patients with MG with ADs was significantly higher than that in patients with MG without ADs (77.0 vs. 52.7%, *p* < 0.05). The proportion of MuSK-positive in patients with MG with ADs was significantly lower than that in patients with MG without ADs (0 vs. 4.8%, *p* < 0.05). No significant differences in other clinical features were found between patients with MG with and without ADs for whom antibody information was available (*p* > 0.05) (Table 3).

**Table 3:** Clinical characteristics between patients with MG with and without ADs for whom antibody information were available.

	**MG with ADs**	**MG without ADs**	***p***
	**(*n* = 74)**	**(*n* = 461)**	
Age at onset (years)	38.7 ± 17.7	40.7 ± 17.9	0.367
Duration (years)	3.7 ± 6.4	2.7 ± 5.0	0.175
Sex			0.077
Male	25 (33.8%)	209 (45.3%)	
Female	49 (66.2%)	252 (54.7%)	
Age at onset (years)			0.504
<50	53 (71.6%)	310 (67.2%)	
≥50	21 (28.4%)	151 (32.8%)	
Thymoma concurrence			0.204
No	59 (79.7%)	333 (72.2%)	
Yes	15 (20.3%)	128 (27.8%)	
Thymectomy			0.299
No	66 (89.2%)	387 (83.9%)	
Yes	8 (10.8%)	74 (16.1%)	
History of MC			0.037^*^
No	74 (100%)	435 (94.4%)	
Yes	0 (0%)	26 (5.6%)	
Family history of ADs			0.000^*^
No	58 (78.4%)	436 (94.6%)	
Yes	16 (21.6%)	25 (5.4%)	
History of allergic diseases			0.437
No	68 (91.9%)	434 (94.1%)	
Yes	6 (8.1%)	27 (5.9)	
Family history of allergic diseases			0.698
No	72 (97.3%)	450 (97.6%)	
Yes	2 (2.7%)	11 (2.4%)	
History of malignancies			1
No	72 (97.3%)	446 (96.7%)	
Yes	2 (2.7%)	15 (3.3%)	
MGFA at onset			0.001^*^
I	57 (77.0%)	243 (52.7%)	
II	17 (23.0%)	195 (42.3%)	
III	0 (0%)	19 (4.1%)	
IV	0 (0%)	3 (0.7%)	
Unknown	0 (0%)	1 (0.2%)	
Antibody status			0.022^*^
AChR-positive	63 (85.1%)	391 (84.8%)	
MuSK-positive	0 (0%)	22 (4.8%)	
Seronegative	11 (14.9%)	48 (10.4%)	

*MG, Myasthenia gravis; ADs, Autoimmune diseases; MC, Myasthenic crisis; MGFA, Myasthenia Gravis Foundation of America; AChR, Acetylcholine receptor; MuSK, Muscle-specific tyrosine kinase*.

* *means p value is < 0.05*.

### Clinical Characteristics Between Patients With MG With 1 and ≥2 ADs

We next compared the clinical characteristics between patients with MG with 1 and ≥2 ADs. No significant differences in clinical features were found between patients with MG with 1 and ≥2 ADs (*p* > 0.05) (Supplementary Table 2).

## Discussion

This study revealed that the percentages of some ADs in our cohort were higher than the background prevalence. Furthermore, patients with MG with ADs presented a lower proportion of previous history of MC. The proportion of MGFA Class I at onset in patients with MG with ADs was significantly higher than that in patients with MG without ADs. The proportion of MuSK-positive in patients with MG with ADs was significantly lower than that in patients with MG without ADs.

It has been demonstrated that MG often coexists with ADs in European or Japanese cohorts. The percentages of concomitant ADs in Norwegian and Danish cohorts have been shown to be 22.9 and 9.4%, respectively (7, 8). A Japanese cohort study reported that associated ADs were found in 28 of 142 patients with MG (19.7%) (9). Moreover, a Swedish population-based study suggested that patients with MG had an increased risk of another AD compared to controls (22.0 vs. 8.9%) (26). A systematic review revealed that the pooled estimate of coexisting ADs in MG was 13% (10). However, few studies have been conducted in Chinese cohorts to date. The percentage of overall ADs in our cohort was 11.6%, which was similar to previous studies. The frequency varies widely across studies, which might be due to the differences in study populations, the ascertainment criteria, and selection bias. Increasing evidence from analysis of human leukocyte antigen haplotypes and genome-wide association studies has revealed shared etiopathogenic factors in many ADs (27). However, the exact mechanisms of comorbid ADs in MG are elusive and require further investigation.

The results of this study suggested that the clinical features of patients with MG with ADs were predominantly female and were younger at onset of MG symptoms. Similar to our findings, studies from Danish, Japanese, and Swedish cohorts also suggested a stronger association among younger and female patients with MG (8, 9, 15). Thymoma-associated MG has an unexpected pattern regarding coexisting ADs. Previous studies have reported a lower percentage of second ADs in thymoma-associated MG than that in non-thymoma-associated MG (28–30). A lower risk for autoimmune comorbidity has also been demonstrated in patients with muscle antibodies against titin and ryanodine receptor, which predicts the presence of thymoma in patients with MG (11, 31, 32). Similarly, we found that the percentage of comorbid ADs in patients with MG with thymoma was lower than that in those without thymoma. However, patients with thymic hyperplasia had a higher frequency of concomitant ADs than those without thymic hyperplasia (9). Few studies have reported the frequency of associated ADs in MuSK-positive MG. We found that MuSK-positive MG was rarely accompanied by ADs; this may be ascribed to the rare occurrence of thymic hyperplasia in MuSK-positive MG, which is highly associated with ADs (33). Due to the limitation of small numbers of MusK-MG patients, more cases should be involved to identify it in future study.

In this study, the most commonly associated conditions in patients with MG were hyperthyroidism and hypothyrosis, both of which were present at a significantly increased percentage compared to the general population. A meta-analysis was performed to determine the prevalence of thyroid disorders in patients with MG, which included 39 studies with 24,927 patients with MG (34). The results suggested that the prevalence of thyroid disorders, hyperthyroidism, and hypothyrosis in patients with MG was 10.1, 5.6, and 2.6%, respectively, which was similar to our results. However, the meta-analysis showed that the prevalence of Hashimoto's thyroiditis in patients with MG was 4.6%, which was significantly higher than 0.6% of our cohort. The lower rate of Hashimoto's thyroiditis in our cohort might be ascribed to the differences in demographic variations and the bias resulting from the cross-sectional nature of this study. A common genetic background may explain the high frequency of autoimmune thyroid disease in MG, and human leukocyte antigens B8 and DR3 have been reported in both disorders (35, 36).

Although thyroid disease has been established as a MG-associated comorbidity, vitiligo has been less frequently reported. We observed a 1.3-fold increase in the frequency of vitiligo in patients with MG compared to the general population. Cruz et al. reported that the percentage of vitiligo in a MG cohort was <1.7% (1 in over 60 patients with MG) (37). Moreover, Kubota et al. found a low frequency (0.5%) of vitiligo in patients with MG (38). Compared to the aforementioned studies, our results are in the middle of the previously observed range. Gill et al. found a significantly higher prevalence of MG (0.2%, 36-fold increase compared to the general population) in a cohort of 1,873 patients with vitiligo (16). A 10-year cross-sectional retrospective study of an urban US population also suggested a significantly higher prevalence of MG in 1,487 patients with vitiligo (39). The higher percentage of vitiligo in patients with MG (or the higher prevalence of MG in patients with vitiligo) suggests that these diseases share a similar underlying pathogenesis. Our results support associations between MG and rheumatoid arthritis or polymyositis, which have also been found in other MG cohorts (11, 40). Our study evidences several new comorbid ADs in patients with MG, including immune thrombocytopenic purpura, autoimmune hemolytic anemia, and autoimmune hepatitis. Although concomitant presentation of these diseases with MG has been reported in rare cases, to our knowledge, these diseases have never been investigated in large cohorts of patients with MG. Although no case was found in the present cohort, it is worth noting that several reports indicated MG may coexist with neuromyelitis optica spectrum disorder (41–43). Previous case reports were mostly complicated with thymoma or thymectomy, indicating an immunological link between the central nervous system and the muscles might exist and be partially due to immune dysregulation or paraneoplastic mechanism. Although aquaporin 4 protein was found to harbor in thymus, the underlying precise mechanism is not clearly understood (44).

In this study, patients with MG with ADs showed a higher frequency of ocular MG at onset. Similarly, the results from an Italian cohort and a Polish cohort suggested that MG associated with autoimmune thyroid diseases had a mild clinical expression, with preferential ocular involvement (27, 45). Ocular MG has a unique link to thyroid disease, which might be ascribed to immunological cross-reactivity against epitopes or autoantigens shared by the thyroid and eye muscles (28, 46, 47). Furthermore, we found that patients with MG with ADs were less susceptible to MC. Moreover, our findings suggested that patients with MG combined with ADs tended to have mild clinical presentation, which is consistent with other studies (28, 29).

This study has some limitations. First, bias is inevitable given that the study is a retrospective single-center study. Second, the study is limited by the lack of longitudinal follow-up data. Therefore, a prospective, multiple-center, follow-up study should be conducted to study comorbid ADs in MG.

## Conclusions

In conclusion, we observed a higher frequency of concurrent ADs in a Chinese MG cohort. Thyroid disease was the most common comorbid AD, while other types of ADs included vitiligo, rheumatoid arthritis, Sjögren's syndrome, psoriasis, idiopathic thrombocytopenic purpura, autoimmune hemolytic anemia, autoimmune hepatitis, and polymyositis. Patients with MG with ADs were predominantly female, younger at the onset of MG symptoms, and had a lower frequency of thymoma. Furthermore, MG combined with ADs tended to have mild clinical presentation.

## Data Availability Statement

The original contributions presented in the study are included in the article/Supplementary Materials, further inquiries can be directed to the corresponding authors.

## Ethics Statement

The studies involving human participants were reviewed and approved by the Institutional Review Board of Huashan Hospital Fudan University. Written informed consent to participate in this study was provided by the participants' legal guardian/next of kin.

## Author Contributions

JS and XH contributed to statistical analysis and drafting of the manuscript. SL and CZ conducted the study design and modified the manuscript. LZ, JX, JS, and YW contributed in collection and validation of the clinical and laboratory data.

## Funding

This work was supported by financial grants from the National Natural Science Foundation of China (Nos. 81803542, 81870988, and 82071410), Shanghai Municipal Science and Technology Major Project (No. 2018SHZDZX01), and ZJLab.

## Conflict of Interest

The authors declare that the research was conducted in the absence of any commercial or financial relationships that could be construed as a potential conflict of interest.

## Publisher's Note

All claims expressed in this article are solely those of the authors and do not necessarily represent those of their affiliated organizations, or those of the publisher, the editors and the reviewers. Any product that may be evaluated in this article, or claim that may be made by its manufacturer, is not guaranteed or endorsed by the publisher.

## Abbreviations

MG: Myasthenia gravis
AChR: acetylcholine receptor
MuSK: muscle-specific tyrosine kinase
ADs: autoimmune diseases
MC: myasthenic crisis
MGFA: Myasthenia Gravis Foundation of America
ITP: Immune thrombocytopenic purpura
AHA: Autoimmune hemolytic anemia
AH: Autoimmune hepatitis.
