# Supplementary material for: Comorbid Autoimmune Diseases in Patients With Myasthenia Gravis: A Retrospective Cross-Sectional Study of a Chinese Cohort
Source: Front Neurol. 2021 Nov 22;12:790941. doi: 10.3389/fneur.2021.790941 (PMC8645996; doi:10.3389/fneur.2021.790941)
Supplement: Supplementary file 1 [file Data_Sheet_1.doc]

**Table S1 Clinical characteristics** **between patients with MG with and without ADs**

|  | MG With ADs  (n=92) | MG Without ADs  (n=704) | *p* |
| --- | --- | --- | --- |
| Age at onset (years)  Duration (years)  Sex  Male  Female  Age at onset (years)  <50  ≥50  Thymoma concurrence  No  Yes  Thymectomy  No  Yes  History of MC  No  Yes  Family history of ADs  No  Yes  History of allergic diseases  No  Yes  Family history of allergic diseases  No  Yes  History of malignancies  No  Yes  MGFA at onset  I  II  III  IV  Unknown  Antibody status  AChR-positive  MuSK-positive  Seronegative  Unkown | 38.1±18.3  3.6±6.6  33 (35.9%)  59 (64.1%)  67 (72.8%)  25 (27.2%)  75 (81.5%)  17 (18.5%)  83 (90.2%)  9 (9.8%)  92 (100%)  0 (0%)  73 (79.3%)  19 (20.7%)  85 (92.4%)  7 (7.6%)  90 (97.8%)  2 (2.2%)  89 (96.7%)  3 (3.3%)  67 (72.8%)  25 (27.2%)  0 (0%)  0 (0%)  0 (0%)  63 (68.5%)  0 (0%)  11 (12.0%)  18 (19.6%) | 40.7±18.1  2.8±5.5  309 (43.9%)  395 (56.1%)  470 (66.8%)  234 (33.2%)  507 (72.0%)  197 (28.0%)  593 (84.2%)  111 (15.8%)  670 (95.2%)  34 (4.8%)  672 (95.5%)  32 (4.5%)  666 (94.6%)  38 (5.4)  690 (98.0%)  14 (2.0%)  681 (96.7%)  23 (3.3%)  382 (54.3%)  293 (41.6%)  25 (3.6%)  3 (0.4%)  1 (0.1%)  391 (55.5%)  22 (3.1%)  48 (6.8%)  243 (34.5%) | 0.213  0.280  0.148  0.287  0.060  0.163  0.025*  0.000*  0.344  0.707  1.000  0.011*  - |

MG: Myasthenia gravis, ADs: Autoimmune diseases, MC: Myasthenic crisis, MGFA: Myasthenia Gravis Foundation of America, AChR: Acetylcholine receptors, MuSK: Muscle-specific tyrosine kinase

**Table S2** **Clinical characteristics between patients with MG with 1 and ≥2 ADs**

|  | MG With 1 ADs (n=84) | MG With ≥2  ADs (n=8) | *p* |
| --- | --- | --- | --- |
| Age at onset (years)  Duration (years)  Sex  Male  Female  Age at onset (years)  <50  ≥50  Thymoma concurrence  No  Yes  Thymectomy  No  Yes  History of MC  No  Yes  Family history of ADs  No  Yes  History of allergic diseases  No  Yes  Family history of allergic diseases  No  Yes  History of malignancies  No  Yes  MGFA at onset  I  II  III  IV | 38.2±18.3  3.3±6.2  32 (38.1%)  52 (61.9%)  61 (72.6%)  23 (27.4%)  67 (79.8%)  17 (20.2%)  75 (89.3%)  9 (10.7%)  84 (91.3%)  0 (0%)  66 (78.6%)  18 (21.4%)  7 (90.6%)  7 (8.3%)  82 (97.6%)  2 (2.4%)  81 (96.4%)  3 (3.6%)  61 (72.6%)  23 (27.4%)  0 (0%)  0 (0%) | 38.0±19.0  6.3±9.8  1 (12.5%)  7 (87.5%)  6 (75.0%)  2 (25.0%)  8 (100%)  0 (0%)  9 (100%)  0 (0%)  8 (8.7%)  0 (0%)  7 (87.5%)  1 (12.5%)  8 (100%)  0 (0%)  8 (100%)  0 (0%)  8 (100%)  0 (0%)  6 (75.0%)  2 (25.0%)  0 (0%)  0 (0%) | 0.982  0.435  0.251  0.100  0.343  1.000  -  1.000  1.000  1.000  1.000  1.000 |

MG: Myasthenia gravis, ADs: Autoimmune diseases, MC: Myasthenic crisis, MGFA: Myasthenia Gravis Foundation of America
